# Caring for critically ill patients outside ICUs due to a full unit

**DOI:** 10.1186/cc14673

**Published:** 2015-09-28

**Authors:** Abimael C Silva, Camila B Oyama, Cintia MC Grion, Eduardo H Rodrigues, Fabiane Urizzi, Lucienne TQ Cardoso, Renata G Oliveira, Thalita B Talizin

**Affiliations:** 1 Universidade Estadual de Londrina, Vila Operária, Londrina, Paraná, Brazil

## Introduction

Advances in medicine allow patients with comorbidities to live longer and the need for intensive care increases. It is not unusual for the supply of critical care beds to not meet the demand. In this scenario the critically ill patient is cared for in the emergency department or in regular hospital wards.

## Objective

To describe clinical and epidemiologic characteristics of critically ill patients treated outside the ICU due to an unavailability of beds.

## Methods

Prospective cohort study of critically ill patients treated in the hospital wards of a university hospital during a 1-year period. All consecutive patients denied ICU beds due to a full unit who were treated by hospital ward staff and daily intensivist physician consultation during the period February 2012-February 2013 were included. Patients were followed until admission to the ICU or cancellation of the ICU bed request. Clinical and epidemiologic data were collected. Invasive procedures and therapeutic interventions were noted. Outcome was observed at hospital discharge.

## Results

Four hundred and fifty-four patients were analyzed. Patients were predominantly male (54.6%) and the median age was 62 (interquartile range (ITQ): 47-73). Median APACHE II score was 22.5 (ITQ: 16-29), median SOFA score was 8 (ITQ: 4-13) and median TISS 28 was 27 (ITQ: 22-30). Reasons for critical care request were respiratory failure (39%), hemodynamic instability (36.3%), neurologic monitoring (14.5%), cardiac monitoring (7.3%) and postoperative care (2.9%). Invasive mechanical ventilation was used in 266 (65.6%) patients, continuous intravenous vasopressors or inotropic drugs for shock treatment were used in 44.9% and intravenous vasodilators in 5.9% of patients. Median time of follow up was 3 (ITQ: 2-6) days, after this time 204 patients were admitted to the ICU and 250 had the ICU bed request cancelled. The motives for waiving critical care were due to clinical improvement in 122 (26.9%) patients, death in 101 (22.3%) patients, decision to withhold treatment due to futility in 25 (5.5%) patients and transfer to another institution in two (0.4%) patients. Hospital mortality was 65%.

## Conclusion

Caring for critically ill patients outside ICU walls was frequent in the study institution. Patients presented a high severity of disease score, had multiple organ dysfunctions and needed multiple invasive therapeutic interventions. Despite receiving intensive care with specialized consultation, these patients present poor prognosis.

